# Impact of Initiating Screening Programs on Referral and Management of Cervical Cancer in Tanzania

**DOI:** 10.1200/JGO.18.00052

**Published:** 2019-07-25

**Authors:** Ami Sedani, Amr S. Soliman, Khadija Msami, Diwani Msemo, Julius Mwaiselage, Kendra Schmid, Crispin Kahesa

**Affiliations:** ^1^ University of Nebraska Medical Center College of Public Health, Omaha, NE; ^2^ City University of New York School of Medicine, New York, NY; ^3^ Ocean Road Cancer Institute, Dar es Salaam, Tanzania

## Abstract

**PURPOSE:**

We investigated the impact of screening rural patients on referral and management of cervical cancer in Tanzania.

**METHODS:**

After reviewing more than 10,000 medical records of patients with cervical cancer who visited Ocean Road Cancer Institute (ORCI) from 2005 to 2014, 108 patients were from the rural communities of Bagamoyo and Chalinze. We abstracted demographic, clinical, and lifestyle factors and linked the data set to databases of all 1,151 patients who visited the Bagamoyo screening clinic from 2011 to 2014 and 1,273 patients who visited the Chalinze screening clinic from 2012 to 2014.

**RESULTS:**

After initiation of the rural screening clinics, difference in duration from diagnosis to prescribed treatment increased from 50.5 to 88 days (*P* = .030), and duration from referral to treatment increased from 38.6 to 101.3 days (*P* = .041). Proportion of patients who received combination chemoradiotherapy increased from 34.3% to 69% (*P* = .001) and completion of treatment decreased from 94.4% to 72.41% (*P* = .002) after initiation of the ORCI screening clinic. Patients who visited Muhimbili National Hospital had significantly shorter periods between referral and prescribed treatment than patients who did not use the Muhimbili National Hospital (mean ± standard deviation, 49.4 ± 128.8 and 112.1 ± 195.31 days, respectively; *P* = .010). Patients who were treated at ORCI had significantly shorter periods between diagnosis and referral to treatment (mean ± standard deviation, 31.4 ± 62.35 and 36.4 ± 121.79 days, respectively; *P* = .005).

**CONCLUSION:**

Future research should focus on investigating barriers to seeking cancer care, benefits of chemoradiotherapy in this population considering the change in prescribed treatment, and time until diagnosis and treatment. Prescription of complex treatments that require more visits to treatment centers may also contribute to decline in completion of treatment.

## INTRODUCTION

Cervical cancer is the most common cancer in women in East and Central Africa. Specifically in Tanzania, the cervical cancer incidence rate is approximately 54 per 100,000, 1.6 times higher than the average rate in East Africa and 3.9 times higher than the global disease incidence rate.^1-5^

The incidence of cervical cancer in developed countries has been reduced by 70% to 90% as a result of screening.^6^ In countries where Papanicolaou test screening is not feasible, other methods, such as visual inspection using acetic acid (VIA) or Lugol’s iodine, have been determined to be effective because of their low cost and instant results.^7-10^ In low- and middle-income countries, fewer than 5% of women have ever undergone cervical screening,^11,12^ because of lack of guidelines, supplies, and qualified staff and anxiety and fear while awaiting results.^13-16^ Also, a majority of screening clinics are located in urban areas.^13^

Studies have investigated the characteristics of women who participate in screening, receive positive VIA results, and undergo immediate treatment. However, only one study has investigated the relationship between referral to a cancer treatment center after cervical screening and loss to follow-up after referral to cancer treatment in Tanzania.^17^ The aims of this study were to examine diagnosis and referral from two rural communities to the main cancer center of Tanzania and investigate the impact of initiating a screening clinic at Ocean Road Cancer Institute (ORCI) on treatment of patients who were referred or not referred from the two communities.

## METHODS

Tanzania is composed of 30 regions, and Bagamoyo is one of the 64 districts located within the Pwani region of Tanzania.^18^ Bagamoyo is divided into 22 wards and 97 villages.^18^ Bagamoyo is approximately 10,000 km^2^ and has a population of approximately 311,740 individuals.^19^ This retrospective cohort study was based on medical records from ORCI, Bagamoyo District Hospital (BDH), and Chalinze Health Centre (CHC).

### BDH

BDH has 125 beds in five wards and is the only government hospital in Bagamoyo.^19^ BDH started VIA screening in December 2011 at the reproductive and child health clinic and is currently open 2 days per week.^19,20^ A majority of women who visit the screening clinic are referred from the HIV clinic.

For this study, demographics available for patients who visited the screening clinic at BDH from December 2011 to December 2014 and were referred elsewhere for additional treatment were collected. Demographics included: age, marital status, HIV status, suspected cancer, and date of screening.

### CHC

CHC is one of the four government health centers in Bagamoyo. CHC is the only other clinic in the district that performs cervical screening (CCS) besides BDH and is approximately 100 km away from BDH. CHC also started CCS in 2012 and is open twice a week. CHC also uses VIA; however, a majority of women who visit the CCS clinic are HIV negative, in contrast to BDH, which targets HIV-positive women. For this study, we included all patients who visited the screening clinic at CHC from December 2011 to December 2014 and all patients who were referred elsewhere for additional treatment.

### ORCI

ORCI is currently the only specialized cancer treatment center in Tanzania and the only place in the country to receive radiotherapy.^21^ Approximately 80% of patients with cervical cancer visiting ORCI are diagnosed at late stages.^22^

As of 2012, ORCI requires that women seeking treatment for cervical cancer at ORCI have their biopsy results before seeing a physician. ORCI also provides CCS (VIA and Lugol’s iodine) and Pap smears and has capability to perform colposcopy with biopsy. The ORCI screening clinic is open 5 days per week.

For patients who visited ORCI in the years from 2005 to 2014 (excluding 2007) and lived in Bagamoyo, their medical record numbers and full names were identified from the ORCI logbooks. The corresponding medical file was then retrieved for each patient, and additional information was abstracted from the medical record. For year 2007, we used a subset from the databases to find patients from Bagamoyo or Chalinze.^17^ The following information was then collected from patients’ medical records: region and village of residence, year of birth and age, date of diagnosis and prescribed treatment, stage at diagnosis, prescribed treatment, completion of treatment, adverse effects of treatment, referral date(s), referral location(s), religion, marital status, highest level of completed education, current occupation, parity, alcohol and smoking history, comorbidities, HIV status (positive, negative, or unknown), contraceptive history, symptoms at time of initial ORCI visit, length of symptoms at time of admission, menopausal status, history of sexually transmitted infections, history of multiple sexual partners, history of polygamy, date of death (if patient died at the treatment center), and referrals and visits to the Muhimbili National Hospital (MNH).^23^

In addition, for women who visited MNH before arriving at ORCI, additional information regarding their hospital stay was collected, including: initial admission date, initial discharge date, admission ward, tests performed during hospital stay, date of biopsy, date patient was given biopsy results, reason for referral (if applicable), referral location(s), and whether the patient had any major complications during her stay.

Treatment was noted as complete if the patient had a note from a treating physician in the record stating completion of treatment, a radiologist and/or oncologist made a note in the patient’s radiotherapy/chemotherapy record that the patient had completed treatment, or a physician made a note in the patient’s record that the patient had come for the annual follow-up or was in remission. If a patient was lost to follow-up, treatment was not listed and was noted as missing in the database.

All variables available for each patient were abstracted. An electronic database was then created and included all collected information.

### Statistical Analysis

χ^2^ or Fisher’s exact test of association was conducted for categorical variables. One-way analysis of variance or Kruskal-Wallis test was conducted for continuous variables. Statistical analysis was performed using SAS software (version 9.3; SAS Institute, Cary, NC). The study was approved by the University of Nebraska Medical Center Health Sciences and Behavioral Sciences institutional review board, Fred & Pamela Buffet Cancer Center and by the ORCI: Academic, Research, Publications and Ethics Committee.

## RESULTS

### Screening Clinics

**FIG 1:**
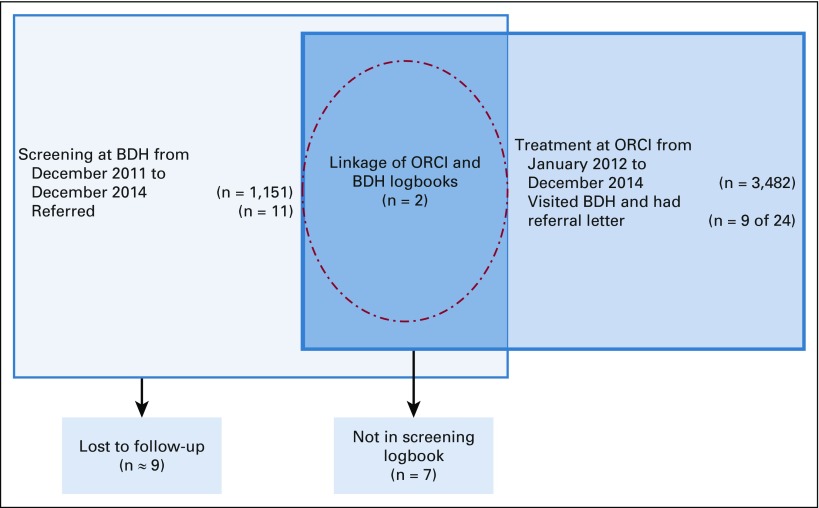
Linkage of Bagamoyo District Hospital (BDH) cervical cancer screening and Ocean Road Cancer Institute (ORCI).

Using the records from January 2012 through December 2014 from ORCI and December 2011 to December 2014 from BDH, the two databases were linked. There were 24 women who visited ORCI from Bagamoyo, nine of whom had BDH referral letters and were recorded as referred from BDH (Fig 1). However, only 11 women in the BDH logbooks were recorded as referred to MNH or ORCI. After linking the ORCI database to the BDH screening clinic database, only two women were in both databases. All names were checked for accuracy.

Using the Chalinze logbooks from December 2011 to December 2014, there were only three women who were referred elsewhere for treatment; however, none of the women in the ORCI logbooks from Chalinze had referral letters from CHC. In addition, none of the women who visited ORCI from Chalinze had CHC listed as the referral location.

### Treatment Hospital

All women visiting ORCI from Bagamoyo and Chalinze were displaying symptoms by the time they arrived at ORCI.

As listed in Table 1, frequency of referral path by region was significantly different. Patients from Bagamoyo primarily visited the ORCI screening clinic (19.51%) or BDH followed by MNH (35.37%; *P* = .001), compared with Chalinze, where a majority (46.67%) of patients went to Tumbi Special Hospital followed by MNH. Other demographics of women from Bagamoyo and Chalinze, such as occupation, education, age, and parity, were not significantly different (Table 1).

**TABLE 1:**
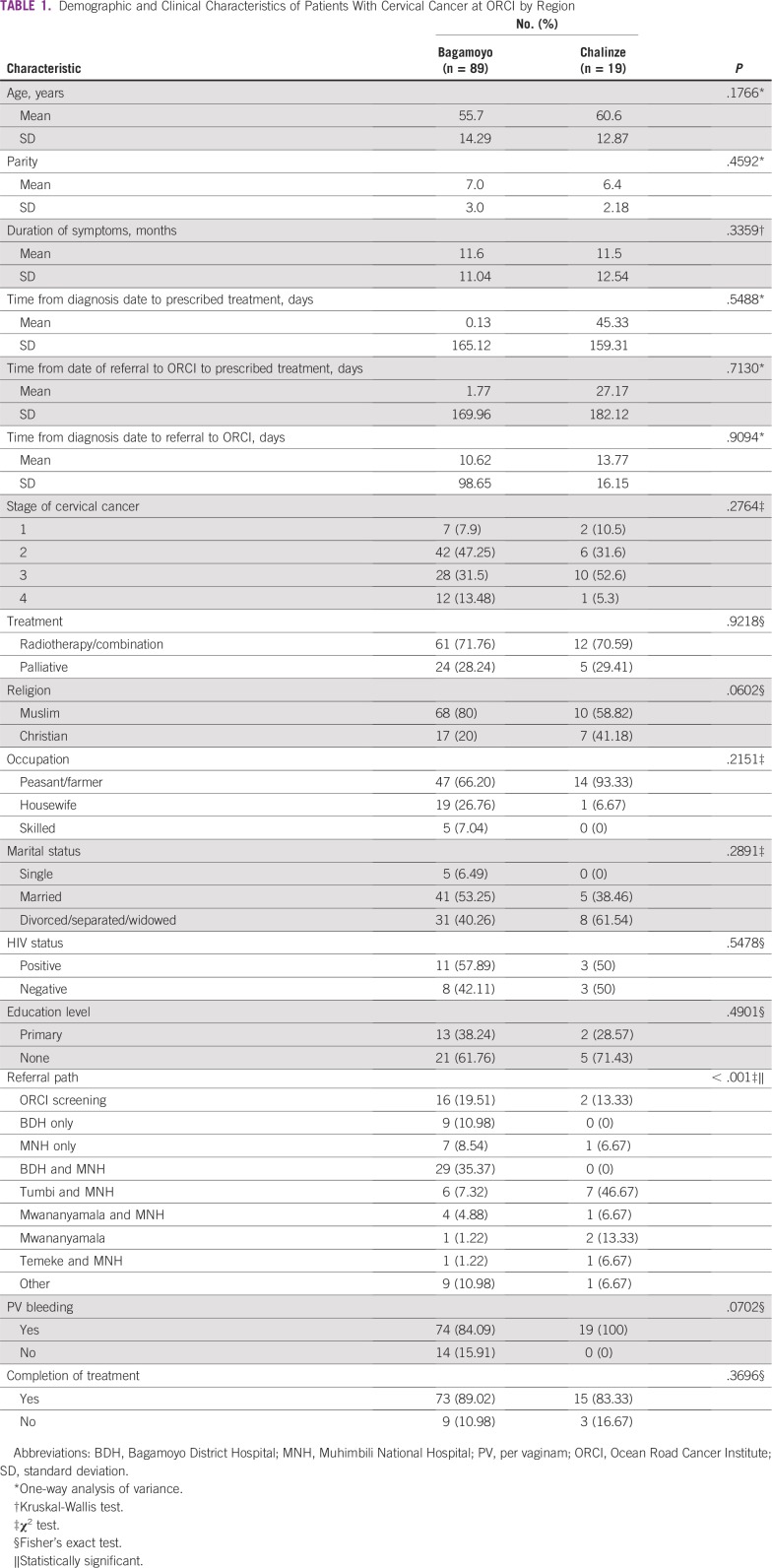
Demographic and Clinical Characteristics of Patients With Cervical Cancer at ORCI by Region

It is important to note that CCS in Tanzania is recommended for women age 30 to 49 years, and the recommended age is lowered by 10 years (starting at age 20 years) for HIV-positive women.^24^ Using the Tanzanian census, we defined the number of women in Bagamoyo and Chalinze who were in the age groups of 20 to 30 and 30 to 49 years and those who were HIV positive during the period of the study.^25^ We also obtained the rate of suspected cancers that should have been referred to ORCI and multiplied the rate by the number of women in the target age groups for whom screening was recommended. This calculation showed that 53 women each from Bagamoyo and Chalinze were eligible for referral during the study period.

Table 2 lists a statistically significant difference regarding mean (± standard deviation [SD]) number of days from diagnosis to date of prescribed treatment (50.52 ± 133.47 and 88 ± 189.31 days for 2005 to 2011 and 2012 to 2014, respectively; *P* = .030). There was also a statistically significant difference (*P* = .041) between the period from referral to ORCI to date of prescribed treatment (mean ± SD, 38.63 ± 154.31 and 101.25 ± 180.10 days for 2005 to 2011 and 2012 to 2014, respectively; *P* = .041). The mean for both differences in dates increased after the implementation of screening clinics. Type of treatment (*P* = .001) and completion of treatment (*P* = .002) were significantly different for the comparison of before and after implementation of screening clinics. There was more radiotherapy (36.7%) and palliative care treatment (28.8%) prescribed before implementation of screening clinics. After implementation of screening clinics, more patients were prescribed combination therapy (69%). A smaller proportion of women completed treatment after implementation of screening clinics than before initiation of the screening programs (72.4% and 94.4% for the two periods, respectively). Stage, age at diagnosis, parity, and duration of symptoms did not differ before and after implementation of the screening clinics. Although more women seemed to be HIV positive over time, rates of patients with HIV-positive status did not differ significantly before and after the screening clinics.

**TABLE 2:**
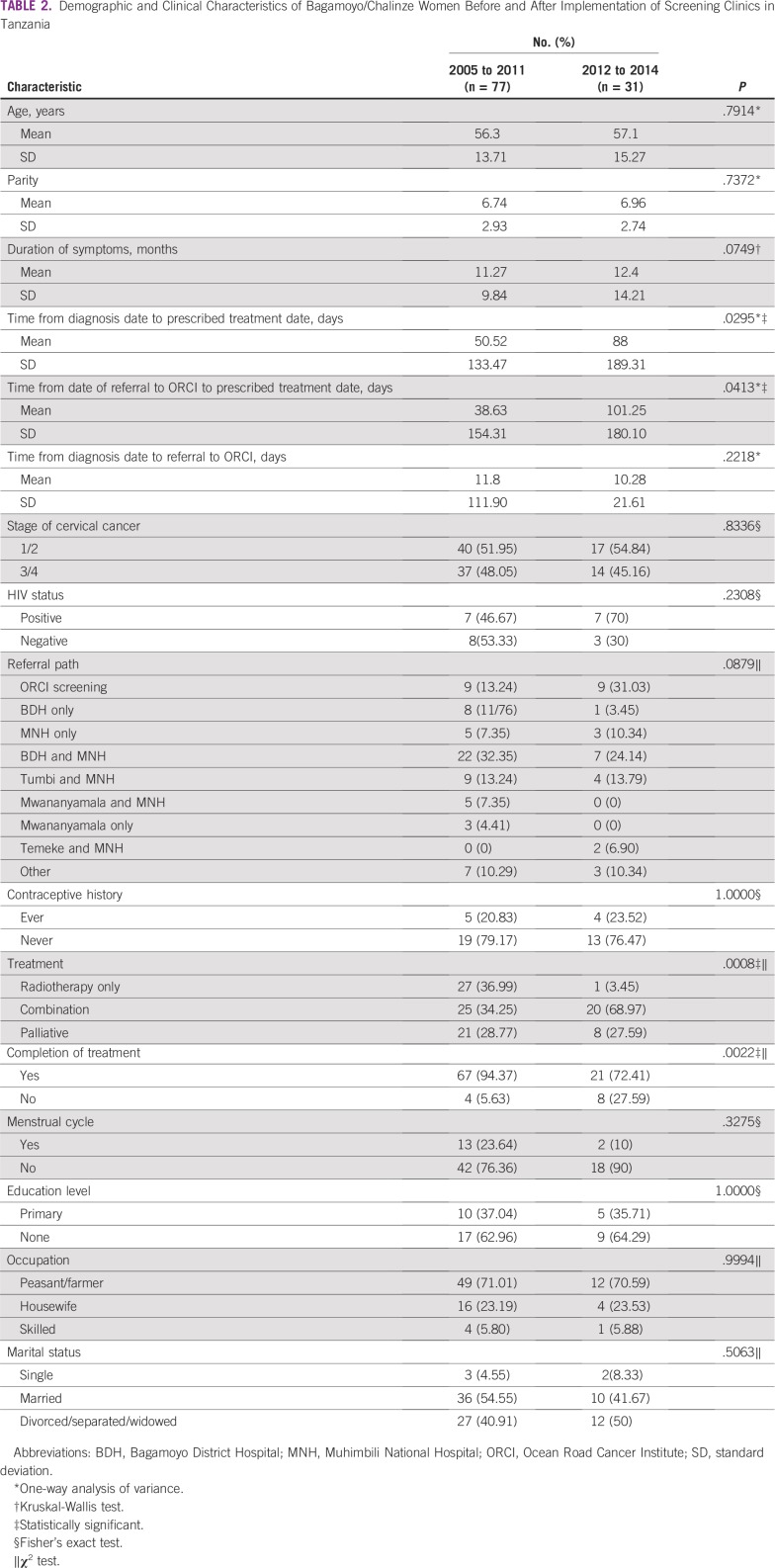
Demographic and Clinical Characteristics of Bagamoyo/Chalinze Women Before and After Implementation of Screening Clinics in Tanzania

Table 3 lists demographics of women who visited ORCI by visit to the referral hospital MNH. There was a statistically significant difference in mean age (*P* = .047) for those who visited MNH compared with those who did not. Patients who visited MNH (mean ± SD, 57.7 ± 13.0 years) were older compared with those who did not visit MNH (mean ± SD, 52.1 ± 13.9 years). There was a statistically significant difference (*P* = .010) when comparing number of days from referral to ORCI until treatment prescription (mean ± SD, 49.4 ± 128.8 and 112.1 ± 195.31 days for those who visited MNH and did not, respectively), and there was a significant difference (*P* = .005) when comparing date of diagnosis (from histology report) with date of referral to ORCI (mean ± SD, 31.4 ± 62.35 and 36.4 ± 121.79 days for those who visited MNH and did not, respectively).

**TABLE 3:**
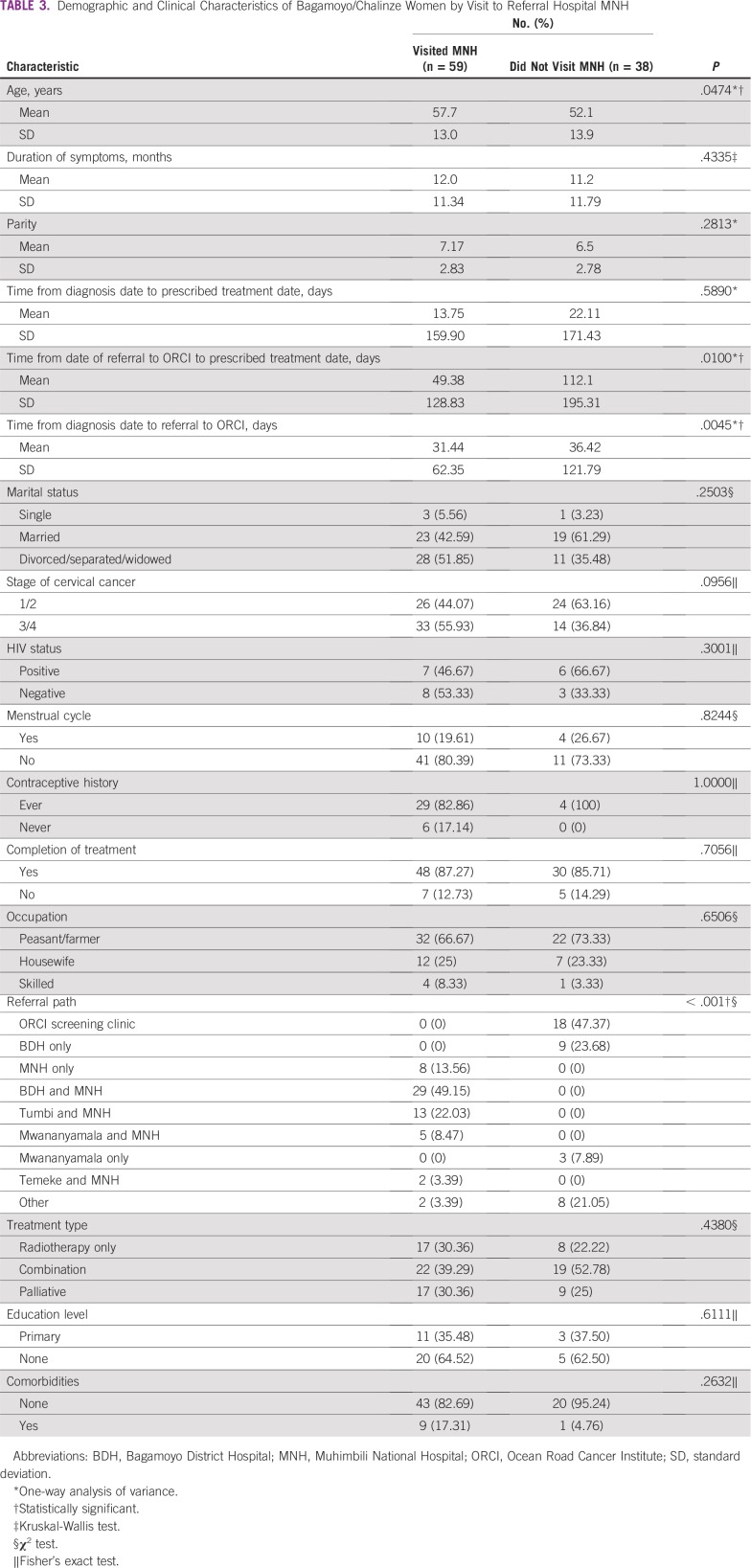
Demographic and Clinical Characteristics of Bagamoyo/Chalinze Women by Visit to Referral Hospital MNH

## DISCUSSION

To our knowledge, this study is the first investigation of the impact of initiating screening programs on referral and management of cervical cancer among rural women in Tanzania. The study revealed the following interesting observations. First, there was a significant difference in self-referral between individuals from Bagamoyo and Chalinze. Patients from Bagamoyo tended to go to ORCI after self-referral to BDH followed by the MNH or ORCI screening clinic, whereas patients from Chalinze tended to go to Tumbi followed by MNH followed by ORCI. Second, after initiation of screening clinics in Tanzania, there were longer periods between diagnosis and prescription of treatment and between referral to ORCI and prescription of treatment. Third, there was a difference in initiation and completion of treatment by time period. Fourth, slightly older women were more likely to visit MNH for their biopsies. Fifth, patients who attended MNH had shorter time periods between referral to ORCI and prescription of treatment at ORCI and between date of diagnosis at MNH to date of referral to ORCI.

Regarding the first observation of difference in referral path, neither BDH nor CHC preform biopsies, so patients with cervical cancer from both sites are required to seek referral to hospitals for biopsy. The difference in referral pattern by region was likely a result of accessibility to diagnostic hospitals. The distance from BDH to ORCI is 67.3 km (1 hour and 38 minutes by car without traffic), and the distance from BDH to MNH is 66.4 km (1 hour and 37 minutes by car without traffic). The distance from BDH to ORCI and MNH is less than 1 km, which would explain the variation in referral path for individuals from Bagamoyo. The distance from CHC to Tumbi Special Hospital is 71.8 km (1 hour and 16 minutes by car without traffic); to Mwananyamala Hospital, 105 km (2 hours and 25 minutes by car without traffic); to MNH, 108 km (2 hours and 33 min by car without traffic); and to ORCI, 113 km (2 hours and 37 min by car without traffic). CHC is closer to Tumbi Special Hospital than it is to the ORCI screening clinic, which could explain the high proportion of patients from Chalinze who were referred from Tumbi. BDH is a district hospital, whereas CHC is a health center; therefore, an intermediate step is required for patients in Chalinze.

Regarding the second observation, after the implementation of screening clinics in Tanzania, there were longer periods between date of diagnosis and prescribed treatment and between date of referral to ORCI and date of prescribed treatment. There was also a difference in treatment and completion of treatment by time period. These results are consistent with our previous study,^17^ which also reported that the proportion of patients completing radiotherapy decreased over time and the volume of patients visiting ORCI increased by 64% over the study period. The increase in number of patients visiting ORCI for cancer treatment since 2007, combined with availability of the same number of radiotherapy machines, has lengthened the waiting time to receive treatment, which could act as a barrier to completing treatment.^23^ However, our previous study found that radiotherapy was the primary treatment prescribed for cervical cancer and that fewer than one fifth of patients received chemotherapy, whereas this study showed increasing trends for combination chemoradiotherapy.

Regarding the third observation, patients who visited the local referral hospital, MNH, for biopsy were slightly older than those who did not visit MNH. The differences in mean age by visit to MNH likely accounted for older women being postmenopausal. Therefore, they may have been more alarmed by abnormal bleeding, as opposed to younger women, who may have mistaken the symptoms for menorrhagia or a sexually transmitted disease and therefore visited the screening clinic instead for diagnosis. In addition, older women can receive free services at the hospital in accordance with the national health plan. Although a majority of patients in Tanzania are eligible to receive free cancer treatment, they must pay for the cost of biopsy when screened at the ORCI screening clinic.^26^ One study found that the mean (± SD) age of patients with cervical cancer in the MNH ward was 48.8 ± 11.1 years; in addition, patient cases and controls had little knowledge of basic symptoms of cervical cancer, which could explain why they visited the hospital instead of undergoing routine screening.^10^ These results are consistent with the findings of studies at Bugando Medical Center in Mwanza, Tanzania, and Kilimanjaro Christian Medical Center Hospital in Kilimanjaro, Tanzania, which found that women seek care at the screening clinics of referral hospitals for abnormal vaginal bleeding rather than undergo cancer screening.^27,28^

Regarding the fourth observation, those who visited MNH had shorter time periods from referral to ORCI to treatment and from diagnosis to referral to ORCI. Patients at MNH were seen at advanced stages of disease and usually remained in the hospital while waiting for their diagnosis and then immediately referred and transferred to ORCI. Patients who were transferred to ORCI from MNH were also seen at ORCI immediately upon arrival. Unfortunately, the average time from biopsy until diagnosis was a minimum of 30 days, whereas patients who visited the ORCI screening clinic waited an average of 7 days.

Strengths of this study included our access to a large number of medical records of most patients with cervical cancer at ORCI, our previous publications on differences in screening and treatment at ORCI, reliable documentation of logbooks for screening and referral at rural clinics, and existence of common variables enabling the linkage of the data sets (eg, name, residence). Limitations of this study included the limited number of days per week during which the screening clinics in Bagamoyo and Chalinze operated, possible unreliability of reporting of permanent addresses, lack of formal computerized tracking of referrals, and inability to generalize these results to screening clinics located farther away.

There was a significant difference in self-referral between individuals from Bagamoyo and Chalinze. After the implementation of screening clinics in Tanzania, there were longer periods between diagnosis and prescribed treatment and between referral to ORCI and prescribed treatment. There was a difference in prescribed treatment type and a decrease in completion of treatment by time period. Slightly older women were more likely to visit the referral hospital MNH before visiting ORCI. Patients who visited MNH had shorter time periods between referral to ORCI to prescription of treatment at ORCI and between date of diagnosis at MNH to date of referral to ORCI. It would be important to compare whether other referral hospitals in Tanzania have similar issues with tracking patients or having patients participate in the screening process.

The national identification system has been initiated in the last few years for government employees, with the intention of its application to all residents in Tanzania in several years. National identification linkage will minimize underestimation of referrals and help track patient treatments and outcomes.

Electronic tracking is becoming feasible and necessary, because transformation of paper medical records to electronic records has been in place at ORCI since January 2017. Also, electronic databases for other diseases, such as HIV, are available at local clinics like Bayagmoyo and Chalinze. Furthermore, standard protocols for treatment are in development in Tanzania, and the results of this study should inform revision and improvement of treatment protocols to suit effective treatment. As increasing numbers of low-income countries establish screening clinics and databases and standardize treatment protocols for patients with cancer that are appropriate for their patient populations and local circumstances, there are opportunities for translating the Tanzanian experience of this research to help with early detection, downstaging, and tailored therapy of cervical cancer in global low-income settings.

Future research should focus on investigating the benefits of chemoradiotherapy in treating cervical cancer in this population, considering the change in prescribed treatment and the time until diagnosis and treatment. Prescription of more complex treatments that require more visits to the treatment center may also contribute to a decrease in completion of treatment over time.
